# Constrictive pericarditis: a known pathology with a new aetiology

**DOI:** 10.1186/1749-8090-10-S1-A263

**Published:** 2015-12-16

**Authors:** Suhaib Ahmed, H ElBasheir, OC Nzewi

**Affiliations:** 1 Department of Cardiac Surgery, Royal Victoria Hospital, Belfast, UK

## Background/Introduction

Pericardectomy remains the only available treatment for constrictive pericarditis.

Historically idiopathic was the main aetiology for CP followed by TB.

In recent years we have seen a change in the aetiology of CP with increasing PCI and surgical interventions.

We reviewed 40 patients who underwent pericardectomy over 10 years period in our institution

## Aims/Objectives

To establish the commonest aetiology of CP in this cohort of patients in light of the relative increase of numbers in our hospital (100% increase in the last 3 years).

To correlate clinical diagnosis with histological diagnosis.

To explore the increasing role of new diagnostic tools like MRI in this known disease.

To detect recurrence rate of CP & need for reintervention.

To compare outcome of pericardectomy in our hospital with published data.

## Method

This was a retrospective study. We reviewed hospital records for all pericardectomy patients over the last 10 years (N = 40) to extract:

1. Aetiology based on histological (when available), intraoperative findings and clinical diagnosis.

2. Follow up information including survival & NYHA post operatively at 3 clinic reviews.

3. Preoperative imaging including Echo, CT and MRI.

4. Interval between the onset of symptoms, diagnosis & surgical intervention.

## Results

The main aetiology in our institution was post cardiac surgery (35%) followed by idiopathic (25%). Post radiation represented only (7.5%).

Clinical diagnosis matched the histological diagnosis in 87.5% of cases.

MRI was the key diagnostic tool in 22.3% of cases.

The interval between onset of symptoms and diagnosis was very variable (3-48 months).

Operative mortality was 5%. Patient with NYHA class III-IV were (17.5%).5 years survival was 85%

## Discussion/Conclusion

A change in aetiology is shown in this limited patients group with post cardiac surgery being the lead one. This was not in the context of surgical closure of pericardium as thought before. The delay in diagnosis of CP post cardiac surgery is an indicator of a diagnosis dilemma. MRI was key in the diagnosis as it implicate a more robust criteria compared to the classical "septal bounce". Further multi-centre studies are required to investigate this new pattern of a known debilitating pathology.

